# NAD^+^-mediated SIRT1–LKB1–AMPK signaling drives lipid remodeling and meat quality differences between Daweishan miniature and Arbor Acres chicken breeds

**DOI:** 10.3389/fvets.2025.1711416

**Published:** 2025-12-31

**Authors:** Hao Wu, Junfeng Luo, Zonghui Jian, Min Yang, Jingying Zhao, Jing Fu, Shixiong Yan, Tengfei Dou, Junjing Jia, Lixian Liu, Zhiqiang Xu

**Affiliations:** 1 Faculty of Animal Science and Technology, Yunnan Agricultural University, Kunming, China; 2 College of Food Science and Technology, Yunnan Agricultural University, Kunming, China; 3 Faculty of Animal Husbandry and Veterinary Medicine, Yunnan Vocational and Technical College of Agriculture, Kunming, China; 4 Yunnan Animal Husbandry and Veterinary Institute, Kunming, China; 5 Institute of Science and Technology, Chuxiong Normal University, Chuxiong, China

**Keywords:** Arbor Acre broiler, breast muscle, Daweishan miniature chicken, chemical composition, NAD^+^, key enzyme, lipid metabolism

## Abstract

**Introduction:**

This study aimed to compare the meat composition, nicotinamide adenine dinucleotide (NAD^+^) metabolism, SIRT1–LKB1–AMPK signaling, and lipid profiles between the slow-growing Daweishan miniature (M1) and fast-growing Arbor Acres (A1) broiler breeds. Methods: Breast muscle samples were collected at 30 days of age (*n* = 6/breed) for chemical analysis, targeted metabolomics, ELISA, and LC–MS-based lipidomics.

**Results:**

Chemical composition showed higher moisture and fat contents in A1 broilers, whereas M1 chickens had greater protein contents (*p* < 0.05). The concentrations of NAD^+^ and its key precursors, including nicotinamide (NAM), nicotinamide mononucleotide (NMN), and nicotinamide riboside (NR), were higher (*p* < 0.05) in the M1 as compared to the A1 chicken breed. ELISA results showed an increased concentration of SIRT1, LKB1, and AMPK in M1 chickens, which was positively associated with NAD+ and its precursors. Lipidomics identified 1,772 lipids across 69 subclasses, with 378 and 310 differentially expressed molecules. The M1 chickens showed downregulation of triglycerides and cholesteryl esters, with mixed regulation of fatty acids. Correlation analysis suggested that NAD^+^-driven SIRT1–LKB1–AMPK signaling associated with lipid catabolism, inhibits lipogenesis, and remodels fatty acid composition.

**Conclusion:**

These results indicate that M1 chickens exhibit enhanced metabolic regulation and protein-rich muscle with distinct lipid remodeling, whereas A1 broilers favor rapid growth and fat accumulation. The study provides mechanistic insight into breed-specific differences in muscle metabolism and meat quality, highlighting NAD^+^ and its signaling axis as key regulators of lipid homeostasis in chicken breast muscle.

## 1 Introduction

Poultry meat is widely consumed animal protein across the globe, owing to its affordability, high nutritional value, and relatively low environmental footprint (1). In the last two decades, breeding practices including intensive selection for rapid growth and high breast muscle yield resulted in the development of international commercial broiler breeds such as the Arbor Acres breed. These birds are characterized by fast growth rates, with better feed conversion efficiency and short production cycles (2). However, it is well established that genetic gains in terms of productivity are also accompanied by metabolic imbalances, excessive fat deposition, and compromised meat quality traits (3, 4). In contrast, local Chinese chicken breeds, such as the Daweishan miniature chicken from Yunnan Province, although have comparatively slower growth rates but retain their resilience, adaptability, and superior meat quality (5), particularly in terms of higher protein density, better flavor, and lower fat contents (6). Therefore, understanding the cellular and metabolic bases underlying these contrasting growth differences is essential for designing breeding programs that balance productivity with nutritional and functional quality.

It is well known that energy metabolism is the fundamental driver in broiler growth and feed efficiency, as it directly determines the allocation of nutrients for muscle protein synthesis versus lipid deposition, ultimately influencing yield and meat quality (7, 8). At the cellular level, NAD^+^ is a pivotal coenzyme system in energy metabolism, functioning as an electron carrier in glycolysis, the tricarboxylic acid (TCA) cycle, and oxidative phosphorylation, while also serving as a substrate for sirtuins and poly-ADP-ribose polymerases (8, 9). The maintenance of NAD^+^ homeostasis is largely dependent on salvage pathways in avian systems, in which precursors such as NAM, NR, and NMN are recycled to sustain intracellular NAD^+^ pools (10). Misbalancing in NAD^+^ metabolism influence not only redox balance but also downstream signaling networks, notably the SIRT1–LKB1–AMPK axis, which governs mitochondrial biogenesis, lipid metabolism, and stress responses (11). Despite the centrality of this pathway in metabolism of the avian species, little is known about how NAD^+^ metabolism differs between fast- and slow-growing chicken breeds and how it contributes to growth performance and meat quality.

Lipid metabolism plays a critical role in influencing muscle growth, membrane structure, and meat flavor of the poultry (12). Lipidomics is a powerful tool for analyzing these muscle lipid-based metabolites and uncovering changes resulting from genetic regulation (13). Recently, LC–MS-based nontargeted lipidomics has become a key method for investigating metabolic mechanisms in chicken muscle. Studies have successfully used this approach to determine how genetics, diet, health, husbandry, breed, and growth rate impact meat quality and lipidomics pathways (14, 15). The studies have highlighted the contribution of glycerophospholipids, sphingolipids, and fatty acid derivatives to membrane remodeling, energy storage, and inflammatory regulation in muscle tissues (16, 17). Moreover, AMPK activation directly influences lipid turnover by inhibiting acetyl-CoA carboxylase and suppressing sterol regulatory element-binding protein 1c-mediated lipogenesis (18, 19). Dissecting the interplay between NAD^+^ metabolism and lipid remodeling in different chicken breeds may therefore reveal mechanistic insights into how growth strategy and muscle quality are metabolically programmed.

In this context, Daweishan miniature chickens and AA broilers provide an ideal comparative model for studying the trade-offs between rapid growth and nutritional characteristics. While AA broilers are optimized for high yield, Daweishan chickens represent a genetic reservoir of traits linked to meat quality, resilience, and adaptive metabolism. Yet, the molecular underpinnings of their divergent muscle growth and metabolic regulation remain poorly characterized. Keeping all this in view, we hypothesized that Daweishan miniature chickens may maintain higher NAD^+^ levels through enhanced salvage pathway activity, which in turn supports stronger SIRT1–LKB1–AMPK signaling and lipid homeostasis. To meet the core objectives, this study integrates multiple analyses of breast muscle from Daweishan miniature chickens and AA broilers. These analyses include comparing proximate composition, characterizing NAD^+^ metabolism and precursor dynamics, investigating the SIRT1-LKB1-AMPK pathway, and conducting lipidomic profiling.

## 2 Materials and methods

### 2.1 Experimental approval, research design, and husbandry practices

Before the research trial, the procedures and protocols of the current study were approved by the Animal Care and Use Committee of the Yunnan Agriculture University (YAU), Kunming, China (No: 20220313; 03/08/ 2022). In this study, a total of 100, zeroday-old chicks (*n* = 50/breed) of Dayunshan miniature (M1) and Arbor Acres (A1) chicken breeds were procured from a commercial hatchery (Changsha Xindacheng Poultry Co., Ltd., China). These chicks were reared at the poultry experimental station of the YAU under identical rearing and husbandry practices. Briefly, the rearing period was divided into two phases, including brooding (0–15 days) and growing (15 days to 50 days). During the brooding phase, chicks were housed in closed cages within a temperature-controlled room at a density of 20–30 birds/m^2^ and fed a starter diet. After that, birds were transferred to step cages at a reduced density of 5–7 birds/m^2^ and fed a grower diet. Feed and water were provided *ad libitum* throughout the experiment. Starter and grower diets were formulated by considering the nutrient requirements specified by the Chinese Feeding Standard of Chicken (NY/T33-2004). The composition and nutritional levels of the diets are presented in Table 1. The temperature was maintained at 32–35 °C for the first week, then reduced by 3 °C weekly. After the fourth week, the room temperature was maintained at approximately 25 °C. The relative humidity was kept at 60–70% for the first 10 days, then at 50–60% during the whole experiment. During the brooding period, 24 h of light were provided for the first 3 days, followed by 23 h of light with gradually reduced intensity. During the growing period, a 10–12 h light/dark cycle was maintained. All birds were subjected to a standard immunization protocol as detailed in Table 2.

**Table 1:** Feed formulation and chemical composition on a dry basis.

Variable	Starter diet	Grower diet
Corn grains (%)	59.13	63.10
Soybean meal (%)	31.46	26.34
Fish meal (%)	3.50	3.50
Soya oil (%)	2.00	3.00
Calcium hydrogen phosphate (%)	1.50	1.50
Limestone (%)	0.70	0.80
Middling flour (%)	0.41	0.46
Methionine (%)	0.08	0.08
Salt (%)	0.22	0.22
Premix^1^ (%)	1.00	1.00
Chemical composition		
Dry matter (%)	89.60	88.80
ME/(MJ/kg)	12.60	12.80
Crude protein (%)	20.55	18.35
Crude fat (%)	5.21	6.42
Ash (%)	5.53	5.12
Calcium (%)	1.05	0.98
Available phosphorus (%)	0.68	0.65
Methionine (%)	0.46	0.45
Lysine (%)	1.35	1.21
Methionine + Cysteine (%)	0.88	0.78
Threonine (%)	0.75	0.71

1 Each kg of premix contained Vitamin A 12,000 IU, Vitamin D3 3,000 IU, Vitamin B1 3.0 mg, Vitamin B2 9.0 mg, Vitamin B6 6.0 mg, Vitamin B12 0.025 mg, Vitamin C 12.6 mg, Vitamin E 18.75 mg, Vitamin K3 2.65 mg, Antioxidants 100 mg, Biotin 0.3 mg, Folic Acid 2.2 mg, Niacin 35 mg, Choline chloride 600 mg, Cobalt 0.3 mg, Copper 12 mg, Iron 50 mg, Iodine 1.0 mg, Manganese 125 mg, Molybdenum 0.5 mg, Selenium 200 mg, Zinc 60 mg.

**Table 2:** An immunization protocol was used in this experiment.

Age (days)	Vaccine	Route of administration	Dose
1	Marek’s disease vaccine	Subcutaneous injection (neck)	1 dose/bird
3	Newcastle disease + Infectious bronchitis (bivalent)	Eye drop	1 dose/bird
12	Infectious bursal disease vaccine	Oral drop	2 doses/bird
20	Newcastle disease + Avian influenza vaccine	Subcutaneous injection (neck)	0.5 mL/bird
25	Infectious bursal disease vaccine	Oral drop	2 doses/bird
35	Fowl pox vaccine	Wing web injection	1 dose/bird
42	Infectious coryza vaccine	Intramuscular injection	0.5 mL/bird
49	Avian influenza inactivated vaccine	Intramuscular injection	0.5 mL/bird
56	Mycoplasma vaccine	Intramuscular injection	0.5 mL/bird

### 2.2 Sample collection

At the 30^th^ day of the experiment, a total of 12 healthy chickens (*n* = 6/breed containing half-male and half-female) were randomly selected for slaughtering and subsequent sampling. Briefly, birds were kept off feed for 12 h with free access to water, and then slaughtered by exsanguination via the jugular vein. Subsequently, the pectoralis major muscle was excised from the central portion, and samples were collected aseptically using autoclaved scissors and forceps. The left-side pectoralis major muscle was dissected, connective tissue was removed, and samples (100 grams/bird) were stored at −20 °C for subsequent proximate analysis. The right-side pectoralis major muscle was similarly sampled (150 grams/bird) and snap-frozen in liquid nitrogen before storage at −80 °C for metabolomic and enzymatic assays.

### 2.3 Chemical compositional analysis

The proximate composition of the breast muscle and feed was determined according to the protocols established by Helrich (20). The meat samples were analyzed (in three replicates) for moisture (method no. 945.15), ash (method no. 967.05), crude protein (Kjeldhal method), and fat contents (Soxhlet method). The moisture, crude protein, fat, and ash contents were calculated by using Equations 1–4, respectively.


$$\text{Moisture}\;(\%)=(\frac{\begin{array}{l} \text{Weight of dried sample}\;(g)\\ -\text{Weight of fresh sample}\;(g) \end{array}}{\text{Weight of fresh sample}\;(g)})X\;100$$ (1)

Crude protein(%)=Nitrogen(%)X6.25 (2)

$$\text{Crude}\;\mathrm{fat}\;(\%)=(\frac{\begin{array}{l} \text{Weight of extracted}\;\mathrm{cup}\\ +\text{sample}\;(g)-\text{Weight of}\;\;\mathrm{cup}\;(g) \end{array}}{\text{Initial weight of sample}\;(g)})X\;100$$ (3)

$$\text{Crude}\;\mathrm{ash}\;(\%)=(\frac{\begin{array}{l} \text{Weight of agnieted crucible}\\ +\text{sample}\;(g)-\text{Weight of}\;\;\text{crucible}\;(g) \end{array}}{\text{Initial weight of sample}\;(g)})X\;100$$ (4)

### 2.4 Enzyme-linked immunosorbent assay

The concentrations of SIRT1, LKB1, and AMPK proteins in breast muscle were quantified using commercial chicken-specific ELISA kits (Jiangsu Jingmei Biotechnology Co., Ltd) according to the manufacturer’s instructions. Briefly, approximately 1 g of tissue was homogenized in 9 mL of normal saline. The homogenate was centrifuged at 3000 × g for 10 min, and the supernatant was collected for analysis. Standards and samples were added to the pre-coated antibody plates, followed by incubation with an HRP-conjugated detection antibody. After washing, the signal was developed with a tetramethylbenzidine substrate, and the reaction was stopped with sulfuric acid. The absorbance was measured at 450 nm by using a spectrophotometer (Varioskan LUX, Thermo Fisher Scientific, USA). The concentrations were interpolated from the standard curve generated for each plate.

### 2.5 Targeted metabolomics analysis of NAD^+^ and its precursors

Chest muscle samples were thawed at 4 °C prior to analysis. Approximately 50 gram tissue sample was transferred into a 2-mL centrifuge tube. A total of 800 μL of 80% methanol (v/v) was added, and the mixture was homogenized at 60 Hz for 5 min. The homogenate was then sonicated at 4 °C for 30 min and subsequently allowed to stand at 4 °C for 1 h. After extraction, samples were centrifuged at 12,000 rpm for 15 min at 4 °C. The resulting supernatant was carefully collected and diluted 1:50 with 80% methanol for LC–MS/MS analysis. Analyses were performed using a Waters Acquity UPLC system coupled with an AB SCIEX 5500 QTRAP mass spectrometer equipped with an electrospray ionization (ESI) source. Chromatographic separation was achieved on a PREMIER BEH Z-HILIC column (1.7 μm, 2.1 mm × 100 mm). The chromatographic conditions were: column temperature 35 °C, flow rate 0.30 mL/min, injecting volume 5 μL and total run time 8 min. Phase A: water containing 10 mM ammonium formate and 0.1% ammonium hydroxide while, Phase B: 90% acetonitrile + 10% water containing 10 mM ammonium formate and 0.1% ammonium hydroxide.

The gradient elution program is shown in Table 3. Chromatographic separation was performed on a Waters Acquity UPLC coupled to an AB SCIEX 5500 QTRAP mass spectrometer. Separation used a PREMIER BEH Z-HILIC column (1.7 μm, 2.1 × 100 mm) at 35 °C with a flow rate of 0.300 mL/min; mobile phase A was water containing 10 mM ammonium formate and 0.1% ammonium hydroxide (v/v) and mobile phase B was 90% acetonitrile/10% water containing 10 mM ammonium formate and 0.1% ammonium hydroxide (v/v). The injection volume was 5 μL, and the total run time was 8 min (gradient below). The QTRAP was operated in positive ESI and scheduled MRM mode with the following source parameters: Curtain Gas 35, Collision Gas (CAD) = 9 (medium), IonSpray Voltage +4,500 V, Temperature 450 °C, GS1 55, GS2 55. Compound-specific MRM transitions, declustering potentials (DP), collision energies (CE) and CXP values used for quantification are listed in the MRM table below. Quantification was performed using external calibration curves prepared in 80% methanol/water and processed with MultiQuant (AB SCIEX); calibration curves with R^2^ > 0.99 were accepted and QC samples were injected regularly during the sequence (Table 4).

**Table 3:** Sample gradient elution conditions.

Time	Flow rate	(B)%	Curve
Initial	0.300	90.0	Initial
1.00	0.300	90.0	6
5.00	0.300	40.0	6
6.00	0.300	40.0	6
7.00	0.300	90.0	6
8.00	0.300	90.0	6

**Table 4:** Mass spectrum acquisition parameters.

Q1Mass (Da)	Q3Mass (Da)	ID	DP (V)	CE (V)	CXP (V)
123	80	NAM	80	20	10
335	123	NMN	95	20	11
255	123	NR	86	15	10
124	80	NA	40	16	10
205	146	Trp	90	18	11
664	524	NAD^+^	25	15	10

### 2.6 Lipidomics analysis

Approximately 60 mg of muscle tissue from each sample was subjected to lipid extraction using a methyl-tert-butyl ether /methanol/water-based method. In detail, samples were homogenized in water, flash-frozen, and lyophilized. The dried powder was subjected to liquid extraction with MTBE/methanol. The upper organic phase was collected, dried under a vacuum concentrator, and reconstituted in isopropanol/methanol (1:1, v/v). Chromatographic separation was performed using the same UPLC system with a gradient elution (Table 5). Mobile phase A was 0.1% formic acid in water, and mobile phase B was 0.1% formic acid in acetonitrile: isopropanol (7:3, v/v). Mass spectrometric data were acquired in both positive and negative ionization modes using an Agilent 6,545 QTOF spectrometer operating in auto MS/MS mode. The mass range was m/z 50–1,300 for MS and m/z 20–1,300 for MS/MS. The ESI source parameters were: gas temperature, 320 °C; drying gas flow, 8 L/min; sheath gas temperature, 350 °C; sheath gas flow, 12 L/min; nebulizer pressure, 35 psig; capillary voltage, 4,000 V (+) and 3,500 V (−).

**Table 5:** Mobile phase elution gradient conditions.

Time (min)	Flow-rate (μL/min)	A (%)	B (%)
0.00	400.00	95.00	5.00
1.50	400.00	95.00	5.00
2.50	400.00	90.00	10.00
14.00	400.00	60.00	40.00
22.00	400.00	5.00	95.00
25.00	400.00	5.00	95.00
26.00	400.00	95.00	5.00
30.00	400.00	95.00	5.00

### 2.7 Statistical and bioinformatics analysis

Raw data were collated in Microsoft Excel 2024, and normality of the data was checked by using qq plots (SPSS software v. 26.0). The data were analyzed by one-way analysis of variance (ANOVA) using SPSS software (v.26.0). The means were compared by adjusting for significant differences at *p* < 0.05. For lipidomics data, raw data files were converted to .abf format and processed using MS-DIAL software (v.4.60) for peak picking, alignment, and annotation. Lipid identification was based on accurate mass (MS1 tolerance: 5 ppm) and MS/MS spectral matching against the LipidBlast database (MS2 tolerance: 0.05 Da). An identification score cut-off of 60 to 80% was applied. Multivariate statistical analysis, including Orthogonal Projections to Latent Structures-Discriminant Analysis (OPLS-DA), was performed using SIMCA (v.14.1). Differentially abundant lipids were filtered based on a variable importance in projection (VIP) score > 1.0 and a *p*-value < 0.05 from ANOVA. These lipids were subjected to KEGG pathway enrichment analysis using MetaboAnalyst 6.0. Data visualization, including volcano plots, heatmaps, and bar graphs, was generated using SRplot, Origin 2021, and GraphPad Prism.

## 3 Results

### 3.1 Comparative meat compositional characteristics

A comparative analysis of breast muscle chemical composition revealed significant differences between the M1 and A1 chicken breeds (Table 6). The moisture content was significantly higher in the A1 breed (74.02 ± 2.94%) compared to the M1 breed (69.10 ± 2.80%; *p* < 0.05). Conversely, the crude protein content was significantly lower in A1 chickens (22.26 ± 2.87%) than in M1 broilers (27.44 ± 2.79%; *p* < 0.05). The crude fat content was also significantly higher in the A1 breed (1.27 ± 0.06%) compared to M1 (0.93 ± 0.07%; *p* < 0.05). No significant difference in ash content was observed between the two breeds (*p* > 0.05).

**Table 6:** Comparative chemical composition of the breast muscle of Daweishan miniature chickens and Arbor Acre broiler breeds.

Nutrient (%)	A1	M1
Moisture	74.02 ± 2.94^a^	69.10 ± 2.80^b^
Crude protein	22.26 ± 2.87^b^	27.44 ± 2.79^a^
Crude fat	1.27 ± 0.06^a^	0.93 ± 0.07^b^
Ash	1.54 ± 0.11	1.55 ± 0.08

A1 represents Arbor Acre broiler breed, while M1 represents Maweishan miniature chicken breed. The different superscripts (a-b) within a row mean a significant difference between groups (*p* < 0.05).

### 3.2 Comparative regulation of the NAD^+^ biosynthesis pathways associated with precursors in the breast muscle

**Figure 1:**
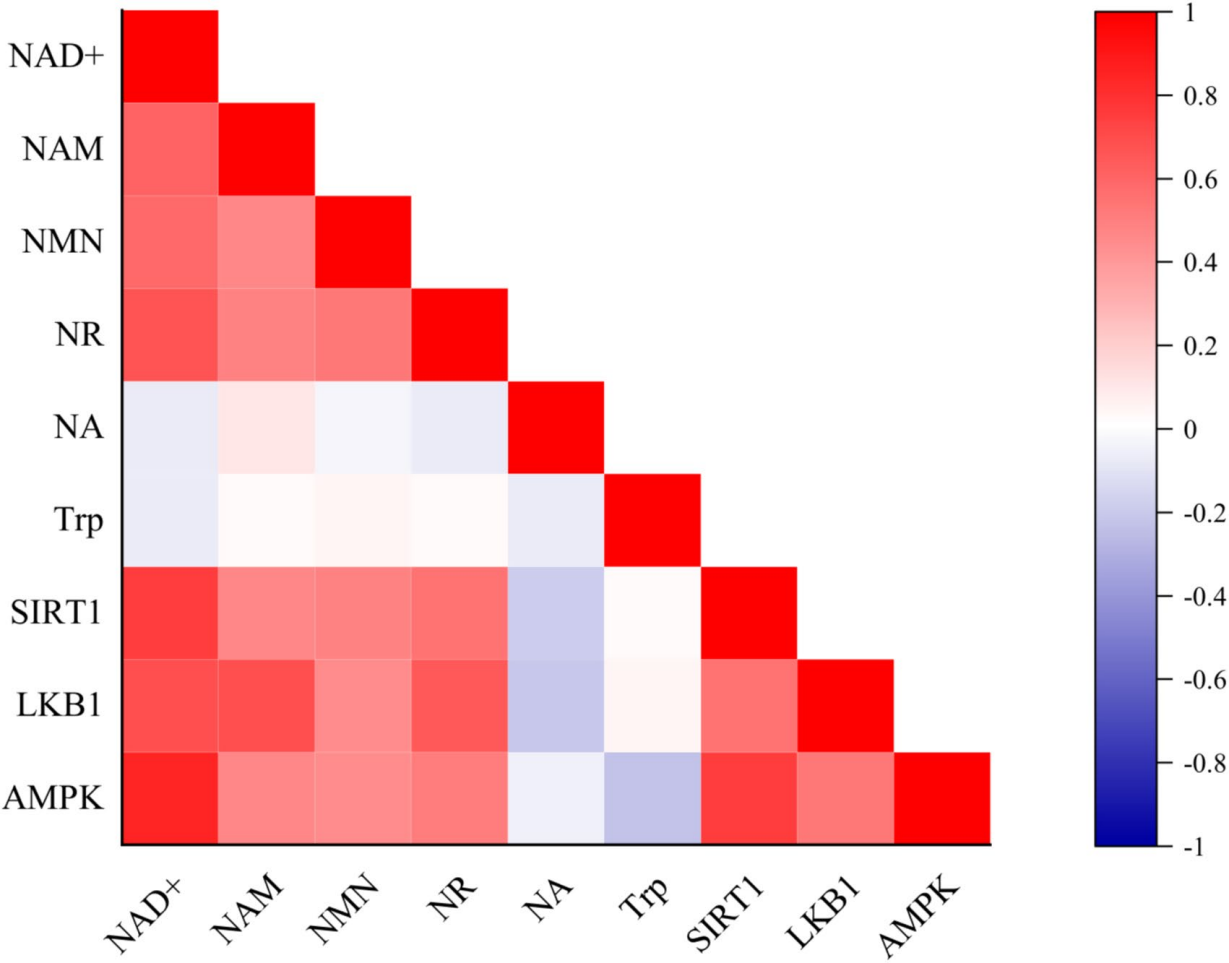
Heat map of correlation analysis of NAD^+^ and its precursors with SIRT1, LKB1, and AMPK enzymes. A network diagram illustrating the interconnectivity and hypothesized regulatory relationships within the NAD^+^ metabolic pathway. Nodes represent measured analytes: NAD+, its precursors (NAM, NMN, NR, NA, Trp), and key enzymes (SIRT1, LKB1, AMPK). The lines (edges) between nodes represent strong positive correlations and co-abundance, with thicker lines indicating stronger relationships. The central positioning of NAD^+^ highlights its role as a hub in this coordinately upregulated metabolic module in the Daweishan miniature chicken breast muscle.

Targeted metabolomics analysis revealed a systemic enhancement of the NAD^+^ salvage pathway in the M1 breed (Table 7). The concentrations of NAD^+^ and its key precursors, including NAM, NMN, and NR, were significantly higher (*p* < 0.05) in the M1 as compared to the A1 chicken breed. However, the levels of nicotinic acid (NA) or tryptophan (Trp) were similar (*p* > 0.05) across the groups. Consistent with elevated NAD^+^ levels, the abundance of major NAD^+^ -dependent enzymes, including SIRT1, LKB1, and AMPK, was higher (*p* < 0.05) in the M1 than in the A1 chicken breed (Table 8). A correlation network analysis showed the strong positive interconnectivity between NAD+, its precursors, and these key metabolic enzymes, suggesting a co-regulated metabolic module that is highly active in the M1 chicken breed (Figure 1).

**Table 7:** Comparative analysis of NAD^+^ and its precursors in breast muscle between Daweishan miniature chickens and AA broilers.

Variables (ng/mL)	A1	M1
NAD^+^	503.63 ± 141.29^b^	689.40 ± 121.18^a^
Nicotinamide	99109.76 ± 531.24^b^	135150.18 ± 967.90^a^
Nicotinamide mononucleotide	731.62 ± 104.51^b^	1022.12 ± 65.85^a^
Nicotinamide riboside	681.30 ± 89.37^b^	989.18 ± 139.87^a^
Nicotinic acid	1509.86 ± 43.25	1698.05 ± 90.30
Tryptophan	13582.52 ± 3325.42	13435.23 ± 1282.08

A1 represents Arbor Acre broiler breed, while M1 represents Maweishan miniature chicken breed. The different superscripts (a-b) within a row mean a significant difference between groups (*p* < 0.05).

**Table 8:** Comparative analysis of three key enzymes in breast muscle between Daweishan miniature chickens and AA broilers.

Enzyme (ng/g)	A1	M1
SIRT1	105.87 ± 7.74^b^	137.74 ± 15.69^a^
LKB1	293.25 ± 22.53^b^	342.51 ± 40.56^a^
AMPK	164.93 ± 14.38^b^	190.15 ± 17.18^a^

The different superscripts (a-b) within a row mean a significant difference between groups (*p* < 0.05).

### 3.3 Multivariate metabolomics analysis of breast muscle

**Figure 2:**
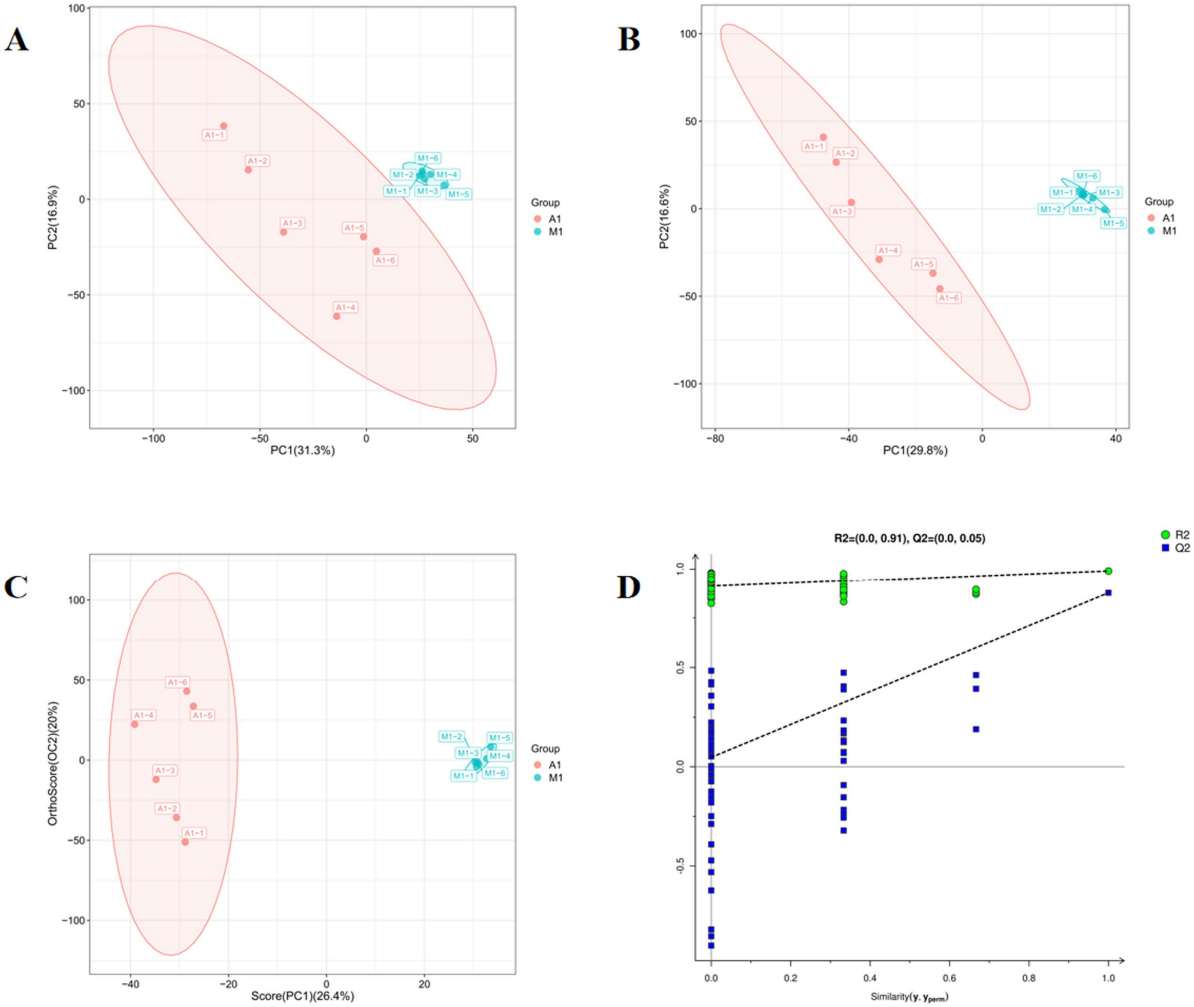
Multivariate statistical analysis of the metabolomics profile in the thoracic muscle of A1 and M1 groups: **(A)** PCA score plot demonstrating the inherent clustering and separation between the two groups; **(B)** PLS-DA score plot demonstrating the maximum separation between A1 and M1 samples; **(C)** OPLS-DA score plot, and **(D)** OPLS-DA Permutation test plot, which filters out non-correlated variation to enhance the visualization of group separation and identify differentially abundant.

Principal Component Analysis (PCA) showed distinct clustering between the A1 and M1 chicken breeds along the first principal component (PC1, 31.3%), indicating significant intrinsic metabolic differences between the two breeds (Figure 2A). A supervised Partial Least Squares-Discriminant Analysis (PLS-DA) model further confirmed this strong separation (Figure 2B). The validity of the model was confirmed by a permutation test, which showed the original model’s goodness-of-fit (R^2^ = 0.91) and predictive ability (Q^2^) were significantly higher than those of the permuted models, thus ruling out overfitting (Figures 2C,D).

### 3.4 Comparative lipdomics profile of breast muscle

**Figure 3:**
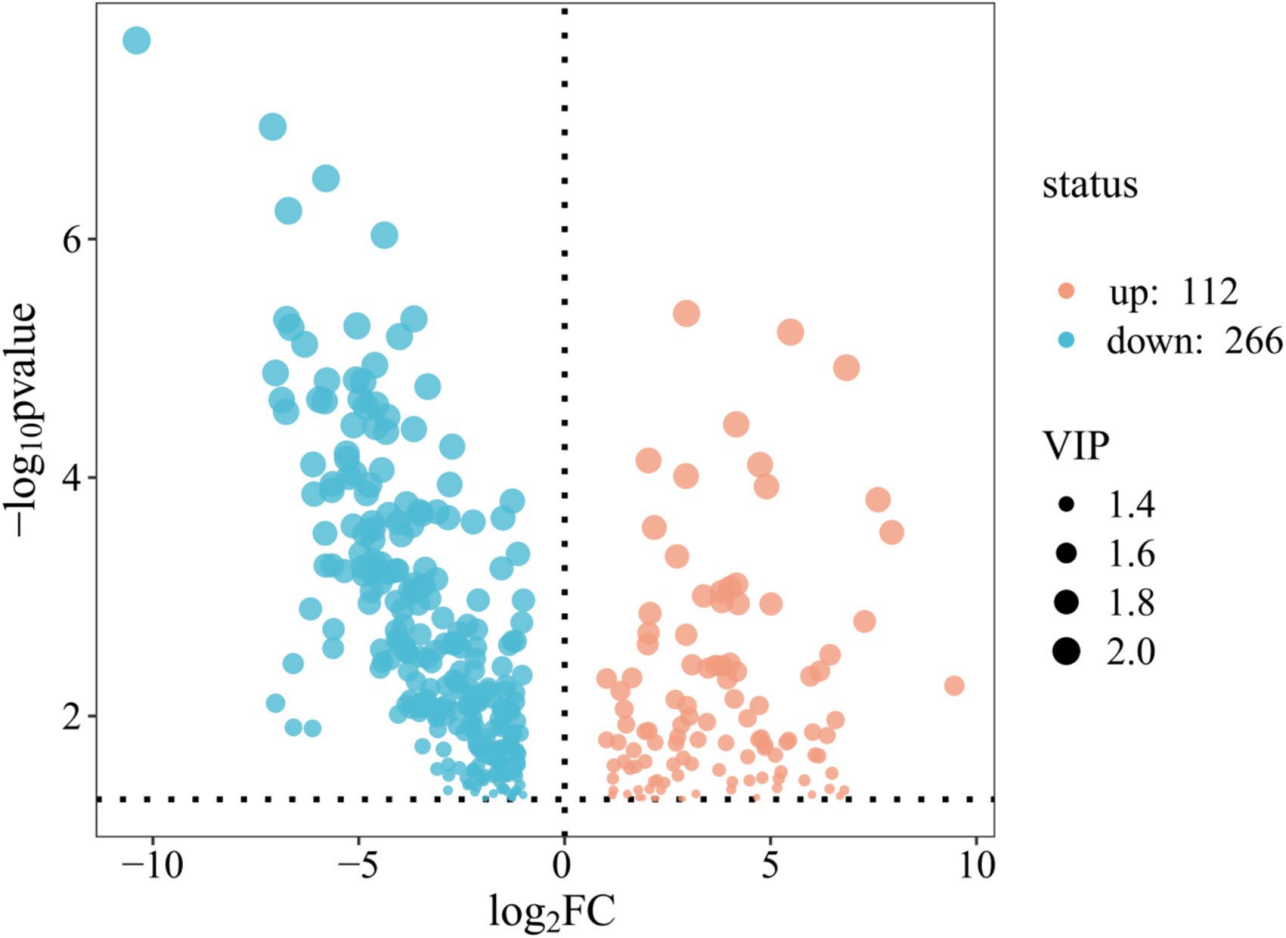
Volcano plot of differentially abundant lipids between A1 and M1 breast muscle. Each point represents an individual lipid molecule. The x-axis shows the log_2_ fold change (M1/A1), and the y-axis shows the statistical significance (−log10 *p*-value). Lipids with a fold change > 0 are upregulated in the M1 group, while those < 0 are downregulated. The gray dashed vertical lines indicate a [log_2_FC] threshold, and the horizontal line represents the *p*-value significance threshold (*p* < 0.05). Points in red (right) and blue (left) represent lipids that are both statistically significant and biologically important (VIP > 1.4).

**Figure 4:**
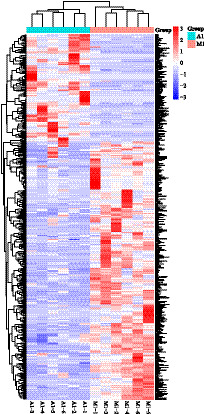
Hierarchical clustering heat map of differential lipids in M1 and A1 groups. Each point represents an individual biological sample. The clear separation with minimal overlap indicates that the overall lipid composition of the breast muscle is fundamentally and significantly different between the two breeds.

**Figure 5:**
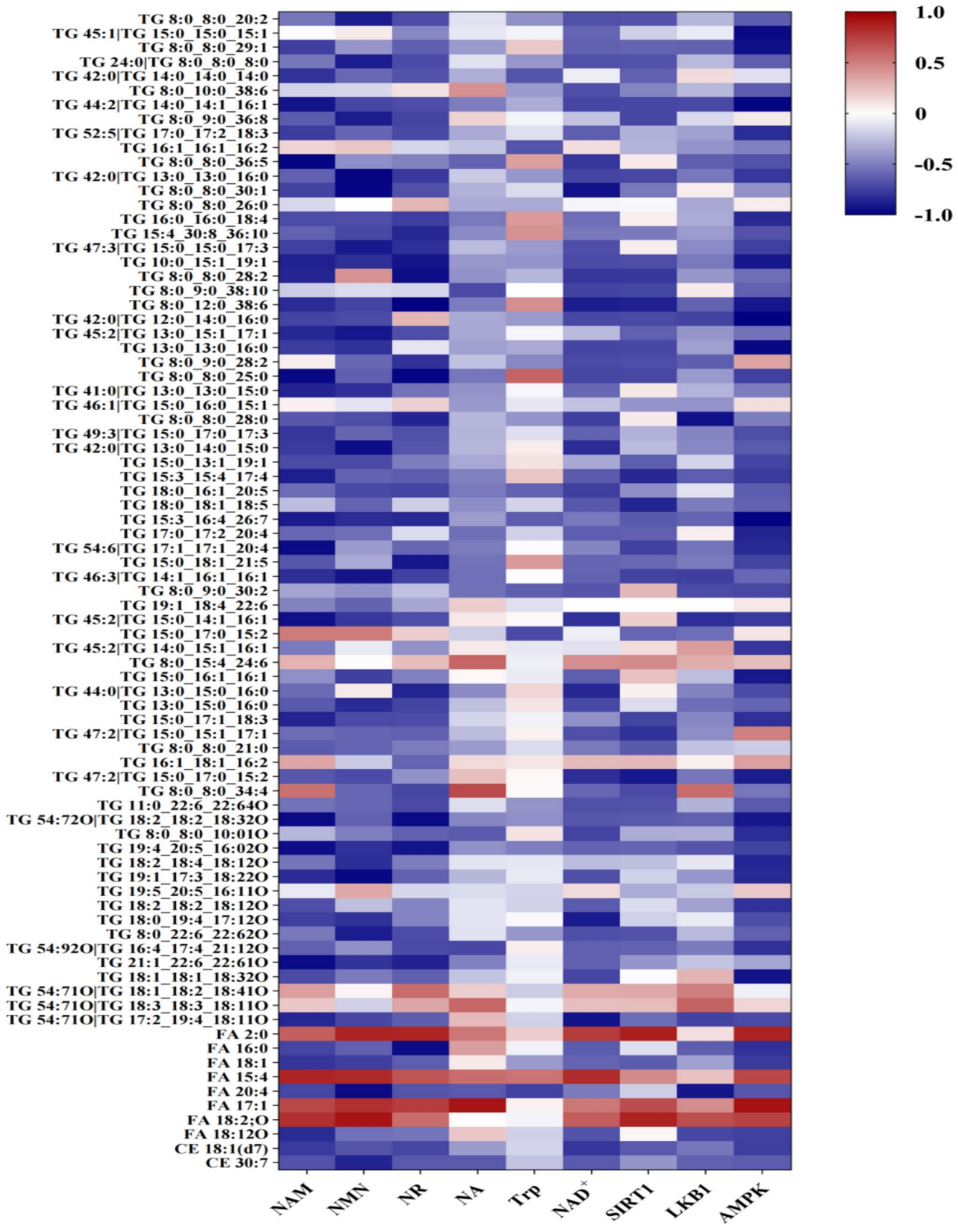
Correlation analysis of NAD^+^, its precursors, key enzymes, and TG, FA, and CE differential lipids in group A1 vs. M1.

Non-targeted lipidomic profiling uncovered a profound rewiring of lipid metabolism between two breeds. A volcano plot analysis identified 378 significantly differentially abundant lipids (VIP > 1.4, *p* < 0.05), with a strong bias toward downregulation in the M1 group. Specifically, 266 lipid species were significantly downregulated, while 112 were upregulated (Figure 3). This widespread alteration resulted in a clear separation of the two breeds, as shown by hierarchical clustering analysis, indicating their overall lipidomes are statistically distinct (Figure 4). The most affected lipid class was triglycerides (TGs); of the 72 TGs identified, 52 were significantly less abundant in the M1 breed, with fold changes ranging from 2.02 to 155.87 (Table 9; Figure 5).

**Table 9:** TG, FA, and CE differential lipid molecules in breast muscle between Daweishan miniature chickens and AA broilers.

Sr. No	Lipid name	Formula	RT (min)	MCR	FC value	*p* value	VIP value	Lipid subclass	Regulation
1	TG11:0_22:6_22:6;O	C_58_H_88_O_10_	27.83	962.67	155.87	0.0016	1.71	TG	↓
2	TG 54:7;2O|TG 18:2_18:2_18:3;2O	C_57_H_96_O_8_	26.97	926.74	114.89	0.0000	1.95	TG	↓
3	TG 8:0_8:0_10:0;1O	C_29_H_54_O_7_	17.57	532.42	110.87	0.0418	1.27	TG	↓
4	TG 8:0_8:0_20:2	C_39_H_70_O_6_	27.95	652.55	102.83	0.0468	1.25	TG	↓
5	TG19:4_20:5_16:0;O	C_58_H_94_O_8_	24.47	936.73	95.56	0.0108	1.5	TG	↓
6	TG18:2_18:4_18:1;O	C_57_H_96_O_8_	25.87	926.74	89.96	0.0301	1.34	TG	↓
7	TG19:1_17:3_18:2;O	C_57_H_98_O_8_	26.86	928.76	87.09	0.003	1.65	TG	↓
8	TG19:5_20:5_16:1;O	C_58_H_90_O_7_	28.22	916.7	86.51	0.0408	1.28	TG	↓
9	TG18:2_18:2_18:1;O	C_57_H_100_O_8_	24.46	930.77	82.99	0.0145	1.46	TG	↓
10	TG18:0_19:4_17:1;O	C_57_H_100_O_8_	22.8	930.77	73.21	0.0041	1.62	TG	↓
11	TG8:0_22:6_22:6;2O	C_55_H_82_O_8_	24.07	888.63	71.97	0.0215	1.4	TG	↓
12	TG 45:1|TG 15:0_15:0_15:1	C_48_H_90_O_6_	28.23	780.71	67.81	0.0212	1.4	TG	↓
13	TG 8:0_8:0_29:1	C_48_H_90_O_6_	28.07	780.71	65.11	0.0136	1.47	TG	↓
14	TG 24:0|TG 8:0_8:0_8:0	C_27_H_50_O_6_	21.46	488.39	64.26	0.0456	1.26	TG	↓
15	TG 42:0|TG 14:0_14:0_14:0	C_45_H_86_O_6_	26.36	740.68	62.66	0.0046	1.61	TG	↓
16	TG 8:0_10:0_38:6	C_59_H_102_O_6_	24.48	929.76	56.59	0.0346	1.31	TG	↓
17	TG 44:2|TG 14:0_14:1_16:1	C_47_H_86_O_6_	25.65	764.68	44.82	0.0000	1.96	TG	↓
18	TG 8:0_9:0_36:8	C_56_H_92_O_6_	28.11	878.72	29	0.0185	1.43	TG	↓
19	TG 52:5|TG 17:0_17:2_18:3	C_55_H_96_O_6_	24.49	875.71	27.81	0.032	1.33	TG	↓
20	TG 16:1_16:1_16:2	C_51_H_90_O_6_	27.97	816.71	27.42	0.0152	1.45	TG	↓
21	TG 8:0_8:0_36:5	C_55_H_96_O_6_	28.1	875.71	26.82	0.0000	1.88	TG	↓
22	TG 42:0|TG 13:0_13:0_16:0	C_45_H_86_O_6_	28.1	740.68	26.43	0.0081	1.54	TG	↓
23	TG 8:0_8:0_30:1	C_49_H_92_O_6_	22.99	794.72	26.19	0.0157	1.45	TG	↓
24	TG 8:0_8:0_26:0	C_45_H_86_O_6_	21.96	740.68	25.33	0.0481	1.25	TG	↓
25	TG 16:0_16:0_18:4	C_53_H_94_O_6_	25.32	844.74	22.47	0.0347	1.31	TG	↓
26	TG 15:4_30:8_36:10	C_84_H_120_O_6_	24.72	1242.94	21.75	0.0219	1.4	TG	↓
27	TG 47:3|TG 15:0_15:0_17:3	C_50_H_90_O_6_	26.91	804.71	21.7	0.0104	1.51	TG	↓
28	TG 10:0_15:1_19:1	C_47_H_86_O_6_	27	764.68	18.58	0.0011	1.74	TG	↓
29	TG 8:0_8:0_28:2	C_47_H_86_O_6_	23.03	764.68	18.01	0.0007	1.76	TG	↓
30	TG 8:0_9:0_38:10	C_58_H_92_O_6_	28.14	902.72	17.97	0.0000	1.91	TG	↓
31	TG 8:0_12:0_38:6	C_61_H_106_O_6_	25.84	957.79	16.49	0.0413	1.28	TG	↓
32	TG 42:0|TG 12:0_14:0_16:0	C_45_H_86_O_6_	26.83	740.68	16.19	0.0036	1.63	TG	↓
33	TG 45:2|TG 13:0_15:1_17:1	C_48_H_88_O_6_	28.16	778.69	16.12	0.0008	1.76	TG	↓
34	TG 13:0_13:0_16:0	C_45_H_86_O_6_	25.95	740.68	15.43	0.0049	1.6	TG	↓
35	TG 8:0_9:0_28:2	C_48_H_88_O_6_	22.91	778.69	15.13	0.0168	1.44	TG	↓
36	TG 8:0_8:0_25:0	C_44_H_84_O_6_	24.42	726.66	14.11	0.0038	1.6	TG	↓
37	TG 41:0|TG 13:0_13:0_15:0	C_44_H_84_O_6_	26.91	726.66	12.61	0.0037	1.63	TG	↓
38	TG 46:1|TG 15:0_16:0_15:1	C_49_H_92_O_6_	28.01	794.72	11.2	0.0039	1.63	TG	↓
39	TG 8:0_8:0_28:0	C_47_H_90_O_6_	24.51	768.71	10.97	0.0112	1.5	TG	↓
40	TG 49:3|TG 15:0_17:0_17:3	C_52_H_94_O_6_	26.99	832.74	10.37	0.0009	1.75	TG	↓
41	TG 42:0|TG 13:0_14:0_15:0	C_45_H_86_O_6_	27.69	740.68	9.43	0.0157	1.45	TG	↓
42	TG 15:0_13:1_19:1	C_50_H_92_O_6_	28.24	806.72	9.11	0.0449	1.27	TG	↓
43	TG 15:3_15:4_17:4	C_50_H_74_O_6_	24.39	793.54	8.53	0.0037	1.63	TG	↓
44	TG 18:0_16:1_20:5	C_57_H_98_O_6_	26.31	896.77	7.71	0.0000	1.88	TG	↓
45	TG 18:0_18:1_18:5	C_57_H_98_O_6_	24.57	896.77	7.11	0.0118	1.49	TG	↓
46	TG 15:3_16:4_26:7	C_60_H_88_O_6_	28.15	927.65	6.52	0.017	1.44	TG	↓
47	TG 17:0_17:2_20:4	C_57_H_98_O_6_	28.17	896.77	4.52	0.0002	1.83	TG	↓
48	TG 54:6|TG 17:1_17:1_20:4	C_57_H_98_O_6_	28.02	896.77	4.2	0.0013	1.72	TG	↓
49	TG 54:9;2O|TG 16:4_17:4_21:1;2O	C_57_H_92_O_8_	26.24	922.71	4.11	0.0000	1.89	TG	↓
50	TG 15:0_18:1_21:5	C_57_H_98_O_6_	24.38	896.77	2.23	0.0462	1.26	TG	↓
51	TG21:1_22:6_22:6;O	C_68_H_106_O_7_	28.19	1052.83	2.02	0.0158	1.45	TG	↓
52	TG 46:3|TG 14:1_16:1_16:1	C_49_H_88_O_6_	28.18	790.69	2.02	0.0048	1.61	TG	↓
53	TG18:1_18:1_18:3;O	C_57_H_100_O_8_	23.58	930.77	0.09	0.0011	1.73	TG	↑
54	TG 19:1_18:4_22:6	C_62_H_98_O_6_	27.45	956.77	0.06	0.0006	1.78	TG	↑
55	TG 8:0_9:0_30:2	C_50_H_92_O_6_	24.51	806.72	0.06	0.0022	1.68	TG	↑
56	TG 45:2|TG 15:0_14:1_16:1	C_48_H_88_O_6_	26.962	778.69	0.05	0.0000	1.91	TG	↑
57	TG 15:0_16:1_16:1	C_50_H_92_O_6_	25.56	806.72	0.04	0.0002	1.84	TG	↑
58	TG 15:0_17:0_15:2	C_50_H_92_O_6_	27.97	806.72	0.04	0.0039	1.63	TG	↑
59	TG 44:0|TG 13:0_15:0_16:0	C_47_H_90_O_6_	28.21	768.71	0.04	0.0001	1.87	TG	↑
60	TG 45:2|TG 14:0_15:1_16:1	C_48_H_88_O_6_	27.66	778.69	0.04	0.0000	1.95	TG	↑
61	TG 54:7;1O|TG 18:1_18:2_18:4;1O	C_57_H_96_O_7_	26.92	910.75	0.04	0.0008	1.77	TG	↑
62	TG 8:0_15:4_24:6	C_50_H_76_O_6_	28.18	790.6	0.04	0.0005	1.79	TG	↑
63	TG 13:0_15:0_16:0	C_47_H_90_O_6_	27.95	768.71	0.03	0.0002	1.83	TG	↑
64	TG 15:0_17:1_18:3	C_53_H_94_O_6_	25.59	844.74	0.03	0.0000	1.97	TG	↑
65	TG 47:2|TG 15:0_15:1_17:1	C_50_H_92_O_6_	26.84	806.72	0.03	0.0002	1.83	TG	↑
66	TG 54:7;1O|TG 17:2_19:4_18:1;1O	C_57_H_96_O_7_	28.14	910.75	0.03	0.0000	1.94	TG	↑
67	TG 54:7;1O|TG 18:3_18:3_18:1;1O	C_57_H_96_O_7_	24.51	910.75	0.03	0.0011	1.74	TG	↑
68	TG 16:1_18:1_16:2	C_53_H_94_O_6_	27.04	844.74	0.02	0.0018	1.69	TG	↑
69	TG 47:2|TG 15:0_17:0_15:2	C_50_H_92_O_6_	27.66	806.72	0.02	0.0000	1.94	TG	↑
70	TG 8:0_8:0_21:0	C_40_H_76_O_6_	26.85	670.6	0.02	0.0000	1.89	TG	↑
71	TG 8:0_8:0_34:4	C_53_H_94_O_6_	23.77	844.74	0.02	0.0000	1.93	TG	↑
72	FA 18:1;2O	C_18_H_34_O_4_	11.45	313.23	43.54	0.0159	1.45	FA	↓
73	FA 18:1	C_18_H_34_O_2_	19.25	281.24	29.21	0.0174	1.43	FA	↓
74	FA 20:4	C_20_H_32_O_2_	16.96	303.23	7.4	0.0495	1.24	FA	↓
75	FA 17:1	C_17_H_32_O_2_	16.98	267.23	4.12	0.0132	1.47	FA	↓
76	FA 15:4	C_15_H_22_O_2_	12.66	233.15	0.38	0.0426	1.27	FA	↑
77	FA 2:0	C_2_H_4_O_2_	0.89	59.01	0.37	0.0216	1.4	FA	↑
78	FA 16:0	C_16_H_32_O_2_	18.69	255.23	0.34	0.0189	1.42	FA	↑
79	FA 18:2;O	C_18_H_32_O_3_	12.52	295.22	0.01	0.0000	2.01	FA	↑
80	CE 18:1(d7)	C_45_H_71_O_2_	23.34	675.67	6.7	0.0149	1.45	CE	↓
81	CE 30:7	C_57_H_90_O_2_	27.93	824.72	3.87	0.0241	1.38	CE	↓

### 3.5 Altered lipids enrich pathways for fatty acid and phospholipid metabolism in breast muscles

**Figure 6:**
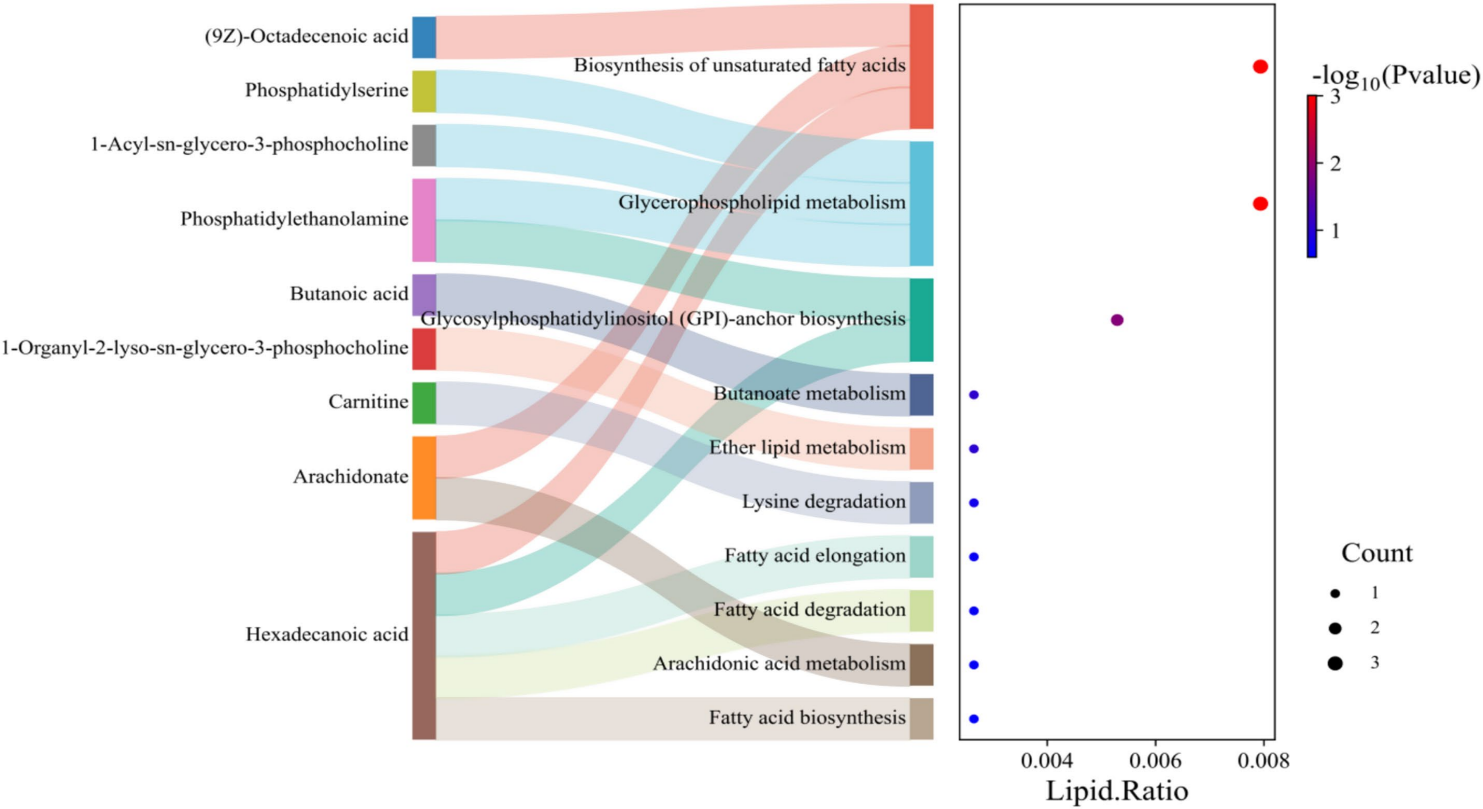
KEGG pathway enrichment analysis of differentially abundant lipids of A1 vs. M1 chicken breed.

To determine the functional impact of the lipid changes, we performed pathway enrichment analysis. This analysis statistically identified the biosynthesis of unsaturated fatty acids and arachidonic acid metabolism as the most significantly altered pathways (highest -log10(*p*-value)). Furthermore, pathways involved in glycerophospholipid metabolism, fatty acid degradation, and fatty acid biosynthesis were also highly enriched (Figure 6). The convergence of numerous significant lipid molecules onto these specific pathways confirms that the metabolic divergence between the breeds has consequential effects on core lipid and energy metabolic processes.

## 4 Discussion

This study elucidates the metabolic divergence between slow-growing Daweishan miniature chickens and fast-growing A1 broilers, revealing a coordinated reprogramming of NAD^+^ metabolism, energy sensing, and lipid handling that underlies their distinct meat quality attributes. Our multi-platform analysis demonstrates that the enhanced growth performance and desirable meat characteristics of A1 broilers are underpinned by a metabolic architecture optimized for energy production and membrane biosynthesis, while M1 chickens exhibit a profile favoring nutrient retention and metabolic regulation.

The proximate composition analysis revealed fundamental differences in muscle architecture. In this study, A1 broilers exhibited higher moisture content. These results suggest that improved water-holding capacity - key traits in meat production (21). Similarly, higher fat contents were found in A1 group, which can be attributed to flavor and palatability. It is well known that higher crude fat content provides abundant flavor precursors in musle through lipid-derived volatile compounds (12), explaining their superior palatability. Conversely, M1 chickens showed higher protein density in this study, indicating a different nutrient allocation strategy that prioritizes nutritional density over rapid growth. These results were augmented by the robust upregulation of NAD^+^ metabolism in M1 broilers. The significantly elevated levels of NAD^+^ and its salvage pathway precursors (NAM, NMN, NR) indicate enhanced flux through this crucial metabolic pathway (22). This expanded NAD^+^ pool supports increased energy metabolism by serving as an essential cofactor for oxidoreductases in glycolysis, the TCA cycle, and oxidative phosphorylation (23), thereby meeting the substantial ATP demands of rapid myofiber hypertrophy. The strong positive correlations between NAD^+^, its precursors, and regulatory enzymes of this study (24) further support the dominance of the salvage pathway in maintaining NAD^+^ homeostasis in avian muscle. Moreover, the coordinated enhancement of the SIRT1-LKB1-AMPK axis in M1 chicken represents a key regulatory mechanism linking NAD^+^ availability to metabolic output. SIRT1, as an NAD^+^-dependent deacetylase, functions as a metabolic sensor that promotes mitochondrial biogenesis and fatty acid oxidation (25, 26). The constitutive upregulation of AMPK suggests a metabolic state perpetually primed for energy generation, efficiently converting nutrients into ATP while inhibiting non-essential anabolic processes (27, 28). This regulatory triad creates a feed-forward loop where AMPK enhances NAD^+^ levels through NAMPT activation, while SIRT1 deacetylates and activates LKB1 to maintain AMPK phosphorylation (26, 29, 30), effectively creating a metabolic environment optimized for growth.

The lipidomic profile provides systems-level validation of this metabolic reprogramming. The enrichment of glycerophospholipid metabolism pathways in this study, reflects the massive membrane biogenesis required for muscle hypertrophy and organelle expansion (31). The altered levels of phosphatidylethanolamine, phosphatidylserine, and various triglycerides indicate heightened investment in structural lipids rather than storage lipids, supporting the extensive tissue remodeling in fast-growing birds (31, 32). The significant changes in sphingolipids, particularly ceramides and sphingomyelins, suggest additional modulation of cellular signaling pathways that regulate growth and metabolism (32, 33).

The functional integration of these pathways is evident in the AMPK-mediated lipid regulation. Through phosphorylation of key enzymes, AMPK promotes TG hydrolysis via ATGL and HSL (34), inhibits FA synthesis by inactivating ACC and SREBP1c (35), and suppresses CE synthesis by targeting HMGCR and SREBP2 (36). This coordinated regulation explains the metabolic shift toward energy production rather than storage in AA broilers, while the higher AMPK activity in Daweishan chickens correlates with their altered lipid profiles and enhanced lipid oxidation capacity.

Notably, the higher activity of the NAD^+^-NAD-NAD-SIRT1-AMPK axis in M1 chickens suggests a more regulated metabolic state that may contribute to their better nutritional profile and potentially enhanced metabolic health. This is consistent with the known role of this pathway in mitigating metabolic disorders (36, 37) and highlights the trade-off between growth efficiency and metabolic regulation in selectively bred poultry. While this study provides comprehensive metabolomic and lipidomic insights, the lack of transcriptional data limits our understanding of the regulatory mechanisms governing these metabolic differences. Future integration of transcriptomics and genomics will be essential to fully elucidate the molecular pathways underlying these breed-specific characteristics.

## 5 Conclusion

Based on results, this study demonstrated that Daweishan miniature chickens and AA broilers exhibited distinct metabolic profiles driven by differences in NAD^+^ biosynthesis, SIRT1-AMPK signaling, and lipid metabolism. Daweishan chickens showed enhanced NAD^+^ salvage pathway activity and AMPK-mediated lipid regulation, resulting in higher nutritional quality but lower fat deposition. In contrast, AA broilers prioritized energy storage and flavor development. These findings provide a metabolic basis for breeding strategies aimed at improving meat quality and nutritional value in poultry.

## Data Availability

The data supporting the findings of this study are available on request from the corresponding authors.
